# Hop, Skip, and Jump: Hydrogen Molecular Transport through Amorphous Polyethylene Matrices Studied via Molecular Dynamics Simulations

**DOI:** 10.1021/acs.iecr.3c02213

**Published:** 2023-11-01

**Authors:** Candice Divine-Ayela, Felipe Perez, Alberto Striolo

**Affiliations:** † Department of Chemical Engineering, University College London, London WC1E 7JE, United Kingdom; ‡ School of Sustainable Chemical, Biological and Materials Engineering, The University of Oklahoma, Norman, Oklahoma 73019, United States

## Abstract

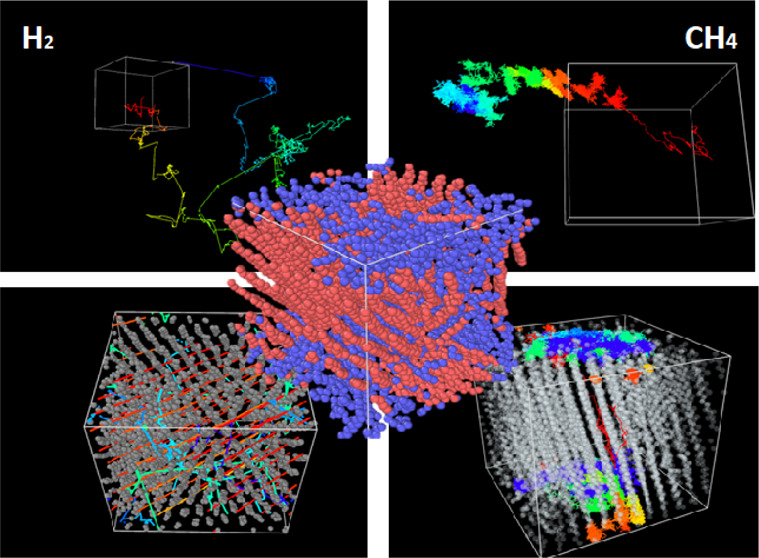

In the pursuit of advancing and diversifying energy technologies for a more sustainable future, the possibilities of hydrogen (H_2_) usage will broaden, as will our understanding of its containment materials. Polyethylene (PE) has a vast assortment of uses and applications, which are growing with demands for alternative energy possibilities. One use of PE liner is as a prime candidate for nonmetallic piping and pressurized type IV storage devices. Such applications require PE to effectively prevent H_2_ transport through containment systems. To study the molecular transport mechanism of hydrogen through polymeric barriers, a system containing hydrogen molecules absorbed within amorphous PE is modeled here using molecular dynamics simulations. The simulations are conducted within a range of temperatures that span the glass transition temperature of amorphous PE. The simulated PE displays bulk density, radius of gyration, and self-diffusion coefficient that are consistent with experimental data. The simulated trajectories are interrogated to study the movement of the guest gas molecules. The results show that the diffusion coefficients increase with temperature, as expected. However, the mobility of the PE chains is found to affect the mobility of absorbed H_2_ molecules to a much lower extent than it affects that of CH_4_ molecules because of the much smaller size of the former than of the latter guest. From a molecular perspective, a “hopping” mechanism is observed, according to which H_2_ molecules hop between one vacant free volume space to another within the polymer matrix, in combination with longer, straight, undisturbed “jumps” or “skips” along directions aligned with regions of ordered PE chains. This suggests that the orientation of the crystallites within the semicrystalline PE matrix affects the H_2_ containment. Implications of these findings toward PE usage as containment material are discussed.

## 1 Introduction

As the world embraces paths toward a more sustainable future, the demand for renewable energy sources increases. To drastically reduce carbon emissions by 2050, low-carbon energy generation options will expand, for example including biofuels and hydrogen fuel combined with carbon capture and sequestration (CCS).^1^ Achieving our future sustainable goals requires adapting and innovating the fuel distribution network. In pipelines, various potential liners are being considered to contain the fuels of the future. One such material, studied for the containment and distribution of hydrogen, is polyethylene (PE).^2,3^

Depending on how hydrogen is produced, it is classified as “gray” (obtained via natural gas or steam methane reforming without CO_2_ capture), “blue” (from natural gas, but incorporating CO_2_ capture), or “green” (via water electrolysis using renewable energy). Gray hydrogen yields 11 tonnes of CO_2_ per ton of H_2_ produced, while producing green hydrogen yields 0.2 tonnes of CO_2_ per tonne of H_2_ or less.^4^ Green hydrogen—though expensive—can be produced entirely using renewable energy in a virtually carbon-neutral process.^5^ Hydrogen-based fuels are expected to spearhead transport-related fueling methods over the next 30 years.^4,6^ As the potential of hydrogen in heating and other applications continues to grow, a quantifiable understanding of its long-term storage and transport challenges is vital.^5,7^ Synergistic to H_2_, biomethane—acquired from anaerobic digestion of biological material or natural gas^3,6^—may potentially mitigate 85% of carbon emission, provided that it can be safely stored and transported without leaks.^8^

Within this context, greater knowledge of materials properties, and their changes when exposed to gases such as H_2_ and CH_4_, and of the mechanisms of gas transport through PE polymeric barrier materials is required to ensure safe storage and efficient transport and to build a widespread commercial infrastructure for gas transport.

Compared to hydrocarbons, hydrogen offers the advantages of being lightweight and offering high energy per unit mass.^9^ Nevertheless, its transport requires complex pressurization and temperature-regulating systems as H_2_ offers low volumetric energy density, and as it can cause embrittlement on metallic materials.^9^ Several materials have been considered for gas-containing purposes, including carbon fiber, graphite nanofiber, steel, multiple metal alloys, and other composites.^2,10^ Nonmetallic piping (NMP) such as reinforced thermoplastic pipes (RTP) and thermoplastic composite pipes (TCP) have been suggested as a lighter, durable alternative to metal pipes. Kane suggested that PE, poly(vinyl chloride) (PVC), or nylon-12 could be suitable for these applications. High-density PE (HDPE) is often considered ideal because it is lightweight, cost-effective, flexible, strong, and resistant to a wide range of substances.^11,12^

A promising method to study gas transport through polymeric barrier materials is offered by molecular dynamics (MD) simulations.^11^ In fact, gas penetrant diffusion in polymers has been investigated via MD over the past few decades. For example, the diffusion of methane in PE polymer matrices has attracted much interest since the 1990s.^13−16^ Making use of the united atom (UA) force field to describe short-chained PE systems, a good agreement was found when describing the structural properties of amorphous low-density PE (LDPE); however, the diffusion coefficient of methane was predicted to be considerably faster than expected. This can be attributed to various causes. For example, the low system densities (∼753 kg/m^3^) simulated at ambient temperature (∼300 K) suggested fully amorphous systems where, experimentally, semicrystalline polymer is likely to be present.^15−18^ More recently, Dutta and Bhatia predicted self-diffusivities of CH_4_, CO_2_, and N_2_ in a membrane made up of short-chain PE molecules to be of the order of 10^–6^ cm^2^/s.^19^ This value reasonably agrees with experimental evidence, where diffusion coefficients in the range of 10^–9^–10^–5^ cm^2^/s have been reported for small gas molecules in PE.^20^ Prior results suggest that to achieve good agreement between simulated and experimental diffusion coefficients, it is important to properly reproduce the density of the polymeric matrix.^21^ In fact, density plays a leading role in PE’s ability to act as a barrier material and it has more of a bearing on the overall penetrant diffusion than that chain length.^22^ Temperature affects the predicted diffusion coefficients of gases in polymeric matrices, with results depending on the molecular structure of the polymer and that of the guest gas molecule.^22^

Smaller molecules like hydrogen have not been studied extensively as guest molecules in polymeric matrices either experimentally or computationally, and gaps exist in the literature concerning the transport mechanism through the polymer matrix, and how such transport, and the resultant polymer–H_2_ interactions, might affect the polymer integrity over time.^2^ Not only is H_2_ small enough to permeate through most materials, but it is also highly flammable.^23^ Hofmann et al. simulated H_2_ diffusion in amorphous polyimides and reported a substantial lack of experimental data as H_2_ was moving too fast to be measured.^24^ The simulated values were up to 5 times greater than expected, which was attributed to the uncharacteristically low system density. In 2018, Yi et al. explored the diffusion of hydrogen and its isotopes in polystyrene. Characteristics of the polymer system such as free volume and density were consistent with experiments; the predicted diffusion coefficients for H_2_ could not be fully validated because of the lack of relevant experimental data.^25^ The landscape is however changing. For example, utilizing two experimental methods, Fujiwara et al. found that hydrogen diffusion in HDPE greatly depends on specific volume and degree of crystallinity—especially at high pressures, where compression can inhibit the diffusion coefficient.^26^ Depending on the H_2_ pressure, slight damage in the PE matrix could be observed, although the mechanisms responsible for these phenomena have not yet been clarified. In 2022, Zheng et al. reported that hydrogen diffusion is regulated by both polymer structure and the quantity of hydrogen molecules present in the bulk PE. In addition, a hopping mechanism was observed.^27^ Zhao et al. found that linear PE was best for containment, as branched and LDPE allowed for an increased likelihood of H_2_ diffusion through the polymer matrix.^28^

This work aims to complement the existing literature and expand our collective mechanistic understanding of the diffusion of H_2_ through PE. A suitable PE model was first developed ensuring that thermodynamic and structural properties are comparable to experimental ones. Using this system, the diffusion mechanism of absorbed H_2_ was studied at the atomic resolution using classical force fields, which are expected to be reliable at the temperatures considered, where tunneling effects are not probable. Chemical reactions are not considered; hence, polymer degradation is not included in the present investigation. The remainder of this manuscript is organized as follows: we first detail simulation methods and algorithms, and we then discuss our main observations, via a comparative assessment of H_2_ vs CH_4_ transport in a PE matrix. We summarize our findings in the context of H_2_ containment using PE liners and suggest possible future research directions.

## 2 Methodology

### 2.1 Polymer Matrix

An amorphous polyethylene (PE) polymer matrix was constructed by implementing the following procedure. Linear *n*-alkanes of varying lengths were considered as prototype PE molecules, with the goal of reproducing the experimental density and molecular mobility of amorphous PE.

Kremer et al. and Fukuda and Kuwajima found polymer lengths (<50 monomer segments) to be a reasonable compromise when computing amorphous properties such as density, volume, and self-diffusion coefficient; achieving results in agreement with experimental data while remaining computationally feasible.^20,29^ Similarly, Meunier and Takeuchi agreed that short-chain polymers (<C_30_ chains) can be viable for simulating some polymeric properties—including diffusion—in addition to models of “infinite length”, as both can provide results comparable to experimental data.^30,31^ After the effect of chain length on the simulation results was tested (results not shown for brevity), the polymer matrix used in this work was constructed using 100 chains of C_50_H_102_ molecules.

The polymer matrices were prepared following Prasad and Börjesson.^32,33^ The bulk was formed by 100 chains of the polymer (C_50_H_102_) placed in a cubic simulation box. Periodic boundary conditions were imposed in *x*, *y*, and *z*-directions with initial cubic dimensions of 25 nm in each direction. An energy minimization routine was conducted using the steepest integrator descent for 5000 steps. After this stage, molecular dynamics (MD) simulations were conducted within the *NPT* ensemble (constant number of particles *N*, pressure *P*, and temperature *T*) at *T* = 900 K, well above the experimental melting point of PE at ∼400 K. After maintaining this for 30 ns before being gradually reduced to the desired system temperatures (100–900 K) over 2 ns and running for a further 10 ns. A time step of 0.5 fs was used. At this point, the density of the systems was extracted to be compared to experimental data in the literature as a function of temperature.^32,33^

### 2.2 Penetrant Gas Molecules: Hydrogen and Methane

To represent H_2_, a rigid diatomic model with an overall neutral charge was utilized, following prior works.^34−36^ Methane (CH_4_) was also simulated to compare transport characteristics between H_2_ and CH_4_ in the polymer matrix and the known experimental literature. CH_4_ was modeled by a united atom (UA) representation consistent with the formalism implemented to simulate PE chains.^14^ As H_2_ solubility in polymers is generally low,^11,35^ the systems were simulated approaching infinite dilution conditions: one gas molecule was placed within the polymer matrix to solely concentrate on the penetrant behavior. This approach was followed to study both H_2_ and CH_4_, even though methane has a higher solubility in PE than H_2_.

### 2.3 Force Fields

Out of 12 force fields, da Silva et al. found that the GROMOS UA—followed by TRAPPE and CHARMM-UA—was able to reproduce experimental density, self-diffusion coefficient, and radial distribution functions of the studied alkane chains on par with the best of the explicit all-atom force fields while remaining computationally efficient.^37,38^ Hence, the GROMOS-UA force field was chosen for this study. To build the polymer chains, UA units were constructed and optimized using Avogadro^39^ and Automated Topology Builder (ATB).^40^ The properties of the PE chains were described by bond length, bond angle, as well as by nonbonded dispersive interactions.^34,41^ Consistent with the UA formalism, the methylene (CH_2_) and methyl (CH_3_) groups of the polymer were described by using the GROMOS-UA force field parameters shown in Table 1. No partial charges were present in the simulated molecules.

**Table 1:** Force Field Parameters Used in the Simulations in This Study^a^

bonded interactions		
	*r*_0_ (nm)	*k*_b_ (kJ/(mol nm^2^))
CH_2_	0.1520	5.430 × 10^6^
CH_3_	0.1510	3.728 × 10^6^

nonbonded parameters	σ (nm)	ε (kJ/mol)
		ε_*ij*_ = (ε_*i*_ε_*j*_)^1/2^
CH_2_	0.40	0.41
CH_3_	0.38	0.87
H_2_	0.23	0.32
CH_4_	0.37	1.26

angles		
	Θ_0_ (deg)	*K*_θ_ (kJ/(mol rad^2^))
C–C	111.0	530.0

a Values from refs (14) and (34).

### 2.4 Algorithms

The MD simulations were carried out using the GROMACS v5.0.4 software package.^42^ The leapfrog algorithm was employed to integrate the equations of motion. The nonbonded interactions were calculated using a Verlet cutoff scheme and potential shifter.^41,43,44^ A cutoff was imposed at 1.4 nm for all interactions. Throughout all of the simulations, the temperature and pressure of the systems were regulated by a Nosé–Hoover thermostat^45^ and a Parrinello–Rahman barostat,^46^ respectively. Ke et al. found that coupling an *NPT* ensemble with the Nosé–Hoover thermostat and the Parrinello–Rahman barostat could accurately reproduce physical properties, achieving results comparable to those obtained using *NVT* and *NVE* ensembles.^47^ Fukada and Kuwajima predicted transport properties for a penetrant in a glassy polymer via implementing either *NPT* or *NVT* ensemble and achieved very similar results.^20^ Hence, the *NPT* ensemble is now widely used to simulate transport.^18,20,48^

The temperatures of 200, 300, and 400 K were chosen to model PE, as PE is expected to transition from glassy to rubber-like in this range.^32,49^ To observe the diffusion and trajectory of the penetrant molecule, Börjesson et al. used a single molecule in a polymer system based on the generally seen low solubility coefficient of a penetrant in a polymeric system. In fact, due to negligible interaction between H_2_ and certain polymers, Kane found very low solubility coefficients with hydrogen in polymeric materials experimentally.^11,33,50,51^ Building on this prior work, a single penetrant molecule was incorporated within the polymer matrix, and the energy minimization was repeated with the same prior conditions. From this point, our simulations were conducted within the *NPT* ensemble with a time step of 0.5 fs over 30 ns at each temperature to quantify the penetrant diffusion and molecular trajectory. The first 5 ns were considered needed to allow for equilibrium to be reached and thus discarded from the final averages of structural properties. The potential and kinetic energies were monitored and plotted as a function of time, and it is verified that they fluctuate about constant mean values to suggest equilibrium has been reached.^30^ The final simulation box was found to be approximately 5 nm in all directions. The results of five independent runs, each starting from different initial positions, were averaged to compute the diffusion coefficient for each temperature.^19^

All simulations were conducted at atmospheric pressure, as pressure effects on diffusion have been reported to be minimal.^30,52^ The diffusion coefficient—*D*—of the different penetrants in PE was determined by computing the mean-square displacement (MSD) of the center of mass, shown in eq 1(15,19,48)
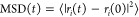
1In eq 1, *r_i_*(0) is the position of the *i*th molecule at time zero and *r_i_*(*t*) is the position of the same molecule at time *t*. The Einstein diffusion relation^53,54^ prescribes a linear slope of the MSD against time for long observation times for regular Brownian diffusion. At these time frames (intervals of ∼2 ns),^49^ there is a degree of proportionality that can be viewed between MSD via the penetrant’s spatial position vs time (*t*) at which point *D* can be extracted. *D* can be estimated from the slope of a linear fit to MSD(*t*) against change in time (Δ*t*) over sufficiently long-time scales according to eq 2(19,49)
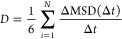
2The diffusion coefficients reported herein were averaged during the *NPT* ensemble over the first 10 ns of each simulation.^19,55^ Following Wang et al.,^56^ MSD curves for each single penetrant molecule were generated and segmented to fit a straight line. When the slope of the fitting line is ∼1, Fickian diffusion is observed, in which case the diffusion coefficient can be determined by using the Einstein relation. To enhance the statistical reliability of our results and follow established procedures, we averaged MSDs of five independent simulation runs over 10 ns to determine the diffusion coefficients for the penetrants.^19,56^

### 2.5 Validation of the PE Model Matrix

As a semicrystalline polymer, PE can exist in three states: glassy, rubbery, and as a viscous liquid.^27,57^*T*_g_ represents the temperature at which a polymer transitions from a rigid, glassy state at low temperatures to a rubber-like state at higher temperatures. When the temperature is increased further, the melting temperature can be reached (*T*_m_), above which the polymer becomes fluid.^58^ The experimental *T*_g_ for HDPE ranges between 140 and 260 K,^59^ while *T*_m_ is between 400 and 528 K at typical semicrystalline and amorphous densities of 970 and 860 kg/m^3^.^58,59^

To validate our model for the PE polymer matrix, we computed the density of the amorphous PE matrix as a function of temperature. The glass transition temperature (*T*_g_) was estimated from the results, and the self-diffusion coefficient was also extracted from the simulations of the PE system without the presence of penetrant molecules. *T*_m_ and *T*_g_ transitions can be quantified by monitoring changes in density trends for the bulk density as a function of temperature.^60,61^

**Figure 1:**
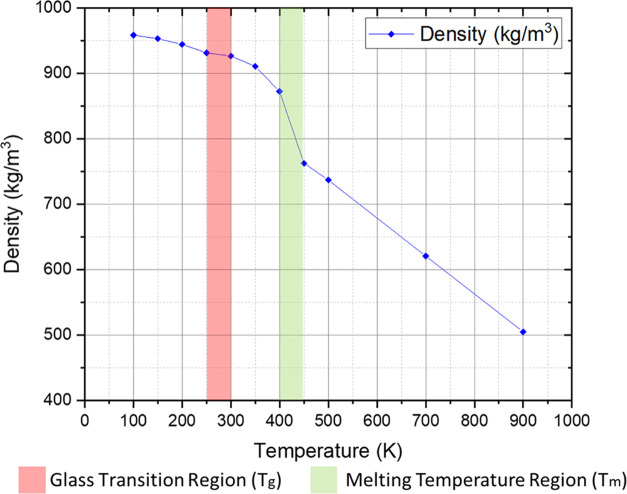
Density of simulated PE system across a temperature range of 100–900 K. The blue line is a guide to the eye.

By plotting the density vs temperature for the simulated PE (Figure 1), we find that the density of the system at 300 K is ∼963 kg/m^3^, *T*_g_ is between 250 and 300 K (in the red region); a very rough estimate for *T*_m_ is 400–450 K (in the green region). These changes can be identified by the change in slope: the plateau at *T*_g_ and the rapid dip at *T*_m_. Of note, because in our model substrates no crystalline region was present at the beginning of the simulations, Tm can only be interpreted as a rough estimate, at which the mobility of the chains increases. Nevertheless, the values extracted from Figure 1 seem to be consistent with experimental values for PE100 with semicrystalline density ranging between 930 and 1270 kg/m^3^.^58,62,63^ Similarly, the self-diffusion coefficient of the polymer chains within the PE matrix (Table 2) was found to be in reasonable agreement with experimental data.^64^

**Table 2:** Self-Diffusion Coefficient of Polymer Matrix at a Temperature Range of 200–500 K

temperature (K)	self-diffusion coefficient (×10^–5^ cm^2^/s)
200	0.14 ± 0.28
300	0.15 ± 0.26
400	0.46 ± 0.38
500	0.93 ± 0.48
experimental	0.48^a^

a Experimental self-diffusion of C_44_ at ∼440 K.^64^

These observations suggest that the model developed for the amorphous PE matrix is reasonable, and it is used in the remainder of the paper to probe the transport behavior of the guest gas molecules.

## 3 Results and Discussion

### 3.1 Penetrant Gas Diffusion Coefficient

The main mechanism of gas diffusion in polymers has been reported to occur by means of “random hops and jumps”, spearheaded by free volume availability. Guest gas dynamics in polymer solids have been typically described based on the transition-state theory.^49,65,66^ The guest gas molecules reside within micro free volume cavities in the polymer matrix, and their movement occurs when new cavities and tunnels bridging said cavities appear. The solute is usually prevented from returning to its original cavity by fluctuations in the polymer chains, which cause channel closing; as a consequence, an effective “hopping jump” motion is observed.^67^ The free volume cavities throughout the polymer matrix are caused by the movement of the polymer chains; their distribution in the matrix varies depending on factors like polymer flexibility, branching, and bulkiness as well as system density, crystallinity, and temperature. These, and other properties influence the permeability of small penetrants within glassy, polymeric materials.^30,68^

As the diffusion of small guest penetrants tends to occur within the amorphous regions of semicrystalline polymers such as PE, the presence of crystallites lowers the diffusion coefficient to different extents depending on the penetrant size.^30,69^ While it is expected that H_2_ will behave like other small guest molecules when absorbed within a polymeric matrix, its very small size and weak interactions with the polymer matrix could lead to unexpected observations. MD is used here to probe such a possibility. Specifically, the trajectories of H_2_ and CH_4_ at infinite dilutions within the PE matrix described in Figure 1 were studied to extract the diffusion at three temperatures: 200, 300, and 400 K. The results are shown in Table 3.

**Table 3:** Simulated Diffusion Coefficient of Hydrogen and Methane in PE at 200, 300, and 400 K

	diffusion coefficient (×10^–5^ cm^2^/s)	
temperature (K)	hydrogen	methane
200	0.30 ± 0.02	0.0006 ± 0.0004
300	0.78 ± 0.03	0.013 ± 0.0007
400	1.49 ± 0.4	0.12 ± 0.006

It helps to compare the simulation results presented here to expectations based on the literature. The diffusion coefficient expected for H_2_ in HDPE is between 10^–5^ and 10^–6^ cm^2^/s^14,26,70^ while the correspondent value for CH_4_ in glassy polymers such as the PE considered here at 298 K and 1 bar is expected to be ∼(1 × 10^–6^)–10^–7^ cm^2^/s.^13,71^ The results in Table 3 are consistent with these expectations, hence confirming the reliability of the models and algorithms implemented. These results relate closely with data obtained computationally and experimentally by Memari^71^ and Michaels and Bixler,^72^ respectively, at dilute conditions.

In addition, the results shown in Table 3 allow us to compare the behavior of H_2_ to that of CH_4_, both at infinite dilution, in amorphous PE. As expected, the diffusion coefficient for H_2_ is much larger than that of CH_4_ at each temperature considered. This is because of the smaller size of the H_2_ molecule compared to that of the CH_4_. As shown in Figure 1, the three simulated temperatures are 200 K, just below the *T*_g_; 300 K, above *T*_g_; and 400 K, near the *T*_m_, respectively, for the PE matrix. It is expected that guest gases diffuse much more slowly as the temperature decreases, and the effect is expected to be more pronounced below the *T*_g_, where the polymer mobility decreases. Because the PE simulated is amorphous, approaching *T*_m_ is not expected to cause significant changes in the diffusion coefficients of guest gas molecules. The results in Table 3 show that for CH_4_, the expectations are matched, with a diffusion coefficient an order of magnitude slower at 200 K than at 300 K, while increasing the temperature from 300 to 400 K increases slightly the diffusion coefficient. On the contrary, the results for H_2_ show a much less pronounced dependency on temperature. In fact, cooling PE below its *T*_g_ value does not significantly decrease the diffusion coefficient predicted for H_2_. This result must be due mainly to the smaller size of H_2_ compared to that of CH_4_, and to a lesser extent, due to the less attractive interactions between H_2_ and PE than those between CH_4_ and PE. This hypothesis is assessed in what follows by investigating the trajectory of the penetrants immersed in the PE matrices.

### 3.2 Transport Mechanism as Revealed by Trajectory Maps

**Figure 2:**
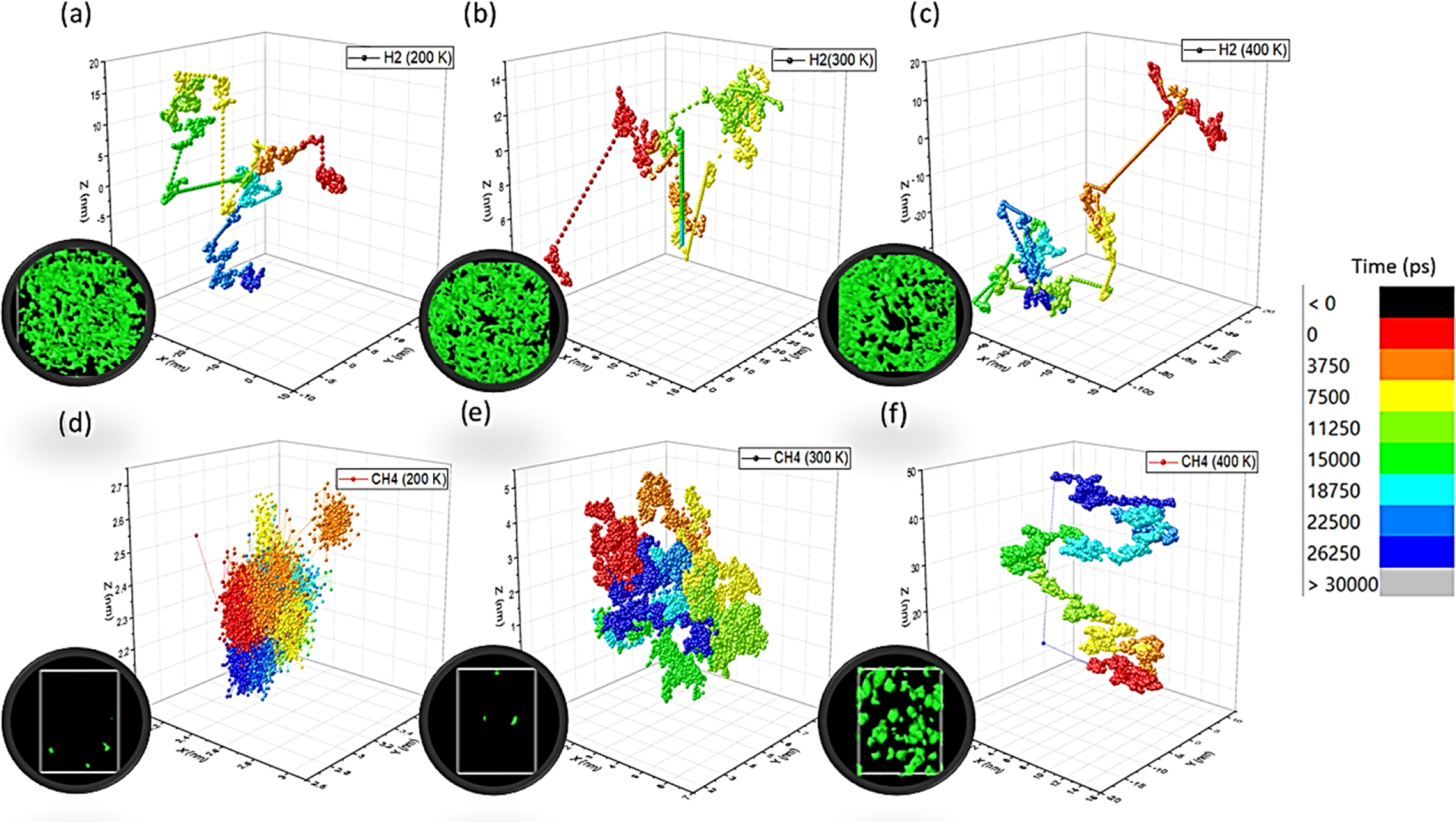
Three-dimensional trajectory path of H_2_ (a–c) and CH_4_ (d–f) in the amorphous PE matrix over time as signified by the color map generated using Origin Lab 2020 software. The inset images reflect the accessible free volume within the respective matrices at *t* = 0 in conjunction with the respective atomic diameter of each penetrant. Note that the scales of the various figures change to reflect the different extents of the gas molecules’ trajectories.

To identify the molecular mechanisms responsible for the differences observed between H_2_ and CH_4_ transport through the PE matrix, we investigate representative trajectories of the guest gas molecules in the temperature range of 200, 300, and 400 K. The results are shown in Figure 2, plotted in three dimensions, as the guest molecules diffuse within 30 ns of observation.^73^

It is evident that at 200 K, CH_4_ is confined within two well-defined molecular-scale cavities within which the absorbed gas can vibrate slightly. Due to the lower thermal energy at 200 K, minimal gyrations occur between the PE chains themselves; however, small microtunnels can be formed over time. In Figure 2d, CH_4_ can be seen to spend time in one cavity over the first 4.75 ns, before making the jump to occupy an adjacent, smaller space and then returning to the initial space. Density fluctuations in the matrix are likely to cause brief shifts while occupying a similar space of approximately 2.4 nm. H_2_ is far less restricted by these boundaries. For example, at 200 K, H_2_ is found to occupy cavities for shorter periods of time compared to CH_4_. The small H_2_ guest molecule exhibits cavity “hopping” jump motions within the first 2.7 ns of the simulated trajectory. This suggests that at this low temperature, H_2_ can be temporarily confined for brief time frames (∼2 ns) before hopping to an adjacent density cavity. The diffusive motions between the longer straight hops and the cavity movement can be perceived as slow motions between polymer chains and intracavity motions due to the small size of the H_2_ molecule.

As shown in Figure 2e, at 300 K, CH_4_ is much more mobile than at lower temperatures. The penetrant occupies slightly larger neighboring molecular cavities for approximately 5 ns at a time. The path is clustered closely together, with each occupied region spanning ∼2 nm and reoccupation of old spaces seen during yellow and lime sections (between 7.5 and 11.25 ns, respectively) as well as during the cyan and dark blue sections (between 18.75 and 30 ns, respectively). This suggests that the amorphous polymeric matrix is producing transient “microtunnels” between cavities more frequently, allowing for short hops, while the previous spaces in the matrix retained a lot of their initial shape and integrity. As opposed to the results just discussed, H_2_ travels freely to a much larger extent than CH_4_ even at 300 K, as shown in Figure 2b. For the first 15 ns, H_2_ moves slowly through the polymer matrix exhibiting similar mobility features described above at 200 K. This is a motion that would be expected in experimental semicrystalline samples—both more rapid “crawling” movement in more mobile amorphized regions alongside slowed hopping movement in more tightly packed or crystalline areas in the polymer. In addition, long straight hopping motions (∼5 nm) can be seen between clustered movements. Due to its small size, H_2_ can move more readily between existing cavities in several directional jumps in comparison to CH_4_. This suggests that despite the ability to diffuse quickly through microtunnels formed by the polymer chains relatively undisturbed, in areas of greater chain mobility, the long straight skipping jump motions can be interrupted by the shifting chains. This results in H_2_ residing briefly within slightly larger cavities or reverting to a more fluid “drifting” motion path through the matrix. At higher temperatures, the latter of the two motions becomes preferred, whereas hopping motion is far more favorable at low temperatures or more fixed structures.

This mechanism appears somewhat more pronounced at 400 K, at which temperature CH_4_ can be seen making more widespread crawling motions through PE and less time occupying small cavities. At these conditions, the gas movement is reminiscent of a random walk path with the now more rapidly moving polymer chains continuously colliding with CH_4_—forcing it to wander aimlessly. Overall, the CH_4_ molecule travels approximately 10 times further than at 300 K (50 nm in the *z*-direction compared to the 5 nm pathway seen prior). Given the relatively large molecular size of CH_4_ (∼3.7 Å), the polymer chain mobility has a great influence on its diffusion path in comparison to H_2_ (∼2.3 Å). At 400 K, H_2_ can still entertain a series of long uninterrupted jumps paired with drifting motions, implying a greater influence by the chain mobility on the trajectory. This suggests that at higher temperatures, when the polymer behaves more fluidly, CH_4_ favors drifting paths composed of small hops between cavities or sweeping motions in which it is carried by the polymer gyrations itself, while H_2_ engages in long “skipping” jumps through microtunnels between distant cavities—only interrupted by the more amorphized areas in the system.^74^

**Figure 3:**
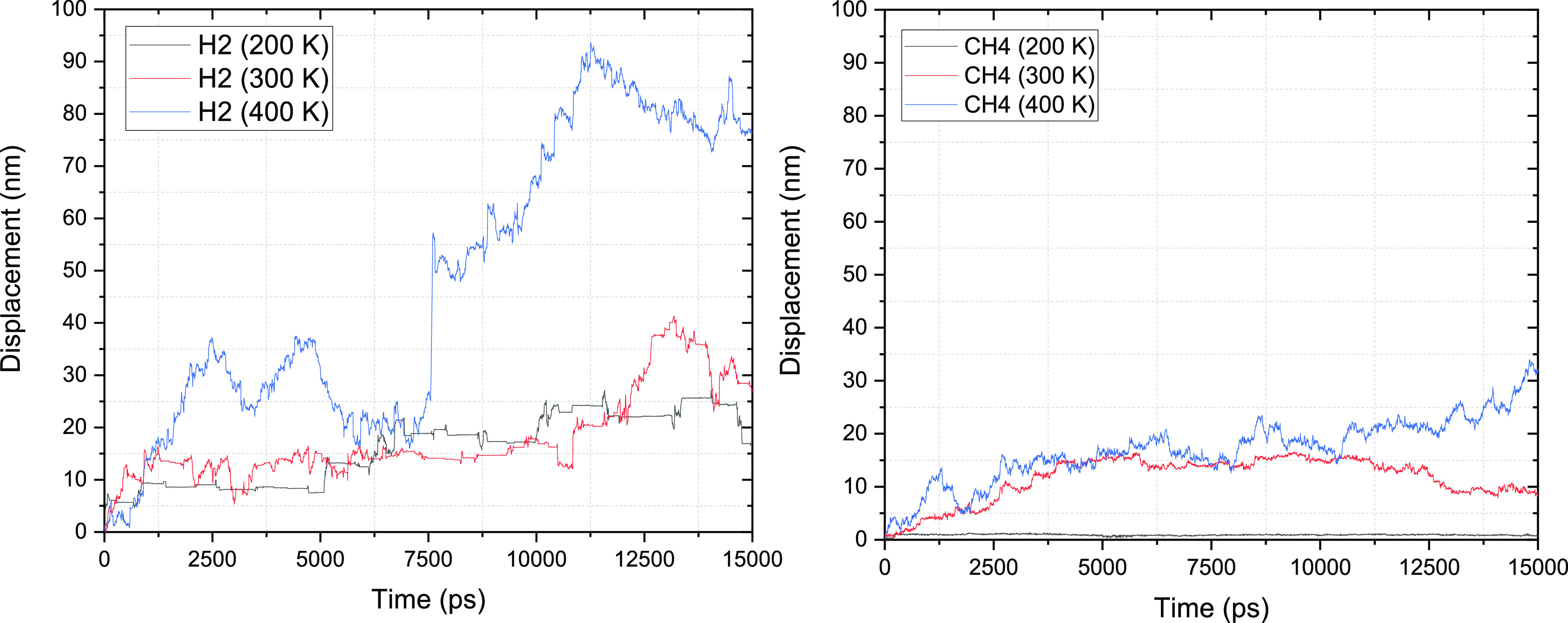
Displacement–time graph for H_2_ (left) and CH_4_ (right) molecules absorbed within the amorphous PE matrix at 200, 300, and 400 K.

To complement the visualization of the trajectories shown in Figure 2, in Figure 3, we show the displacement of one H_2_ and that of one CH_4_ molecule over the first 15 ns of the simulations. At lower temperatures, both molecules exhibit a limited range of motion. H_2_ makes small, sharp movements in addition to slightly larger jumps of around 5 nm at a time, whereas CH_4_ remains stationary. The H_2_ jumps come to abrupt plateaus before resuming movement, which suggests a hopping mechanism in which the molecule moves quickly into microcavities formed due to density fluctuations within the polymer matrix. H_2_ can be seen very briefly contained at the flat peaks of the trajectory, suggesting it meets an abrupt stop when in contact with a less mobile polymer matrix; when this happens, it is likely that H_2_ engages in a new path instead of drifting with the polymer motions. This suggests that though H_2_ can move at low temperatures, it is likely unable to disturb the properties of the polymer matrix, a result due to both H_2_ small molecular size and its weak interactions with PE. At 300 K, the polymer chains have more freedom of motion than below *T*_g_. In the case of H_2_, this yields more frequent and longer jumps (∼10 nm), while evidence of very brief residences in density cavities (∼150 ps) can also be seen. In the case of CH_4_, instead of quick sharp hopping motions, shorter closely linked movements can be viewed. This is much more reminiscent of more fluid diffusive motions, suggesting that as the chain is granted more flexibility, the CH_4_ follows predominantly the motions of the surrounding chain itself. This is possibly due to its size-limiting options for travel through the microcavities that the hydrogen is taking advantage of. While at 400 K, H_2_ appears to utilize both long jumps and liquid-like diffusion among the polymer chains in addition to large jumps interrupted by sudden chain movement. In tandem with Figure 2, the results shown in Figure 3 also support the ability of H_2_ to move quickly via large hops. Additionally, rather straight “skip-like” paths can be viewed before being halted by the matrix on contact or resulting in short intracavity movement. CH_4_ favors much shorter abrupt hops and much more likely to be influenced by the polymer structure itself and follow the chain movement allowing for much more predictable habits of diffusion and containment.

**Figure 4:**
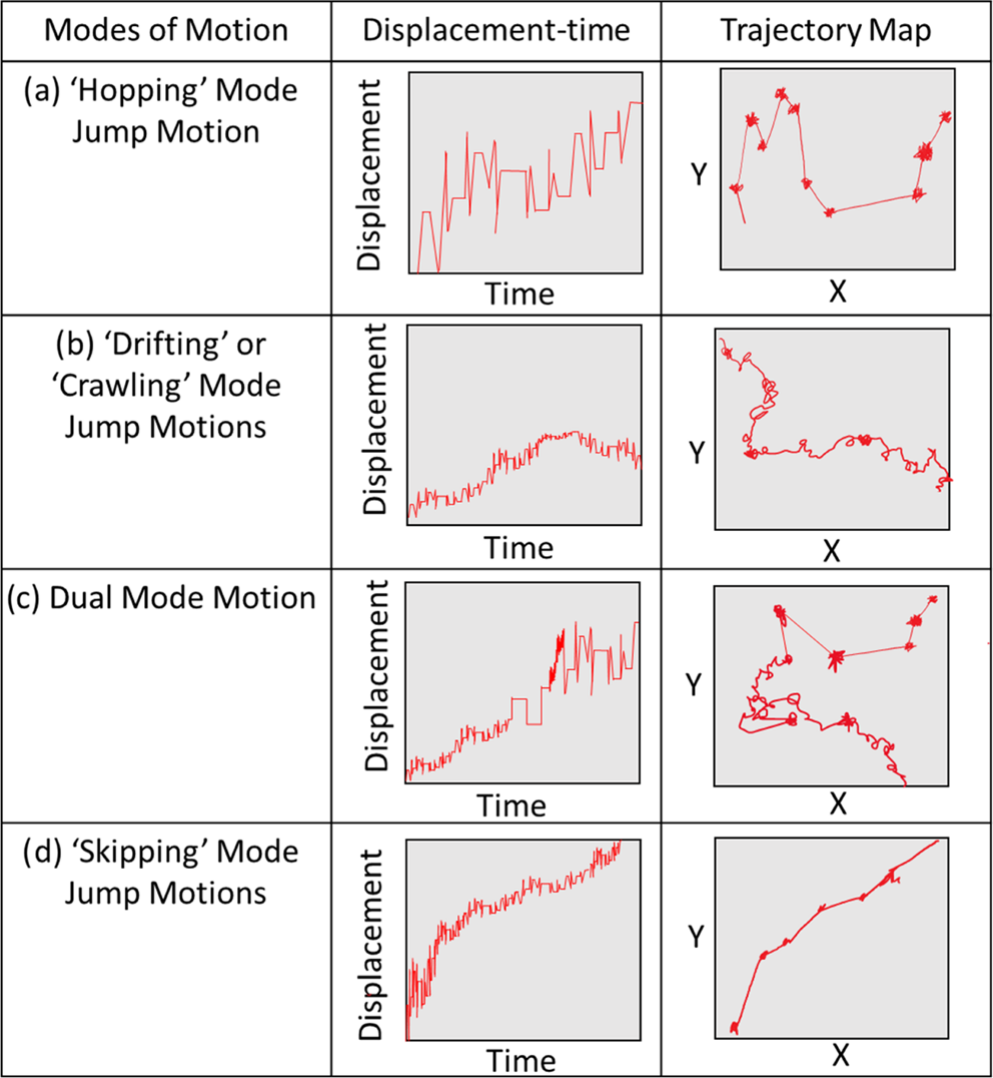
Types of diffusive motion expected of penetrant molecules in polymer matrices and how they manifest in simplified examples of displacement–time graphs and two-dimensional (2D) trajectory maps.

Three prominent mechanisms of motion of penetrants within polymers were projected by Reynier and Sun: crawling or drifting diffusion mode, jumping diffusion mode, and dual diffusion mode (a combination of the two former mechanisms).^75,76^ Differences among the various mechanisms are described schematically in Figure 4. All of these mechanisms can be perceived when compared to results shown in Figures 2 and 3 at various temperatures.

Hopping jump mode (Figure 4a) involves short jumps through microcavities in the polymer separated by periods of cavity movement contained within the polymer low-density regions (i.e., cages). This is typically seen in systems with high densities, lower temperatures, or with larger guest molecules.

Drifting or crawling (Figure 4b) jump motions can occur where the penetrant travels randomly across the polymer matrix through free volume within—often drifting with the chain movement resembling a “random walk” or “polymer chain walking” motion.^77^ When there is a need for quick diffusion of the penetrant at higher temperatures, high molecular weight, or chain mobility, this type of diffusion is frequently utilized.

At certain conditions, both modes can be viewed simultaneously, as seen in Figure 3 for H_2_ at 300 K. An additional mode of transport was identified here, which we identify as “skipping”. Skipping motions (Figure 4d) are composed of multiple movements allowing for longer undisturbed jumps. This behavior can be viewed by H_2_ at every temperature, whereas CH_4_ utilizes hopping motions at 200 K and drifting and dual motions at higher temperatures. This suggests that molecular size plays a key role in the choice of jump that the penetrant utilizes for movement.

### 3.3 Statistical Analysis: Jumps, Skips, and Hops

Statistical analysis can be conducted over the movement of the guest gas molecules confined within the polymeric matrices because diffusion has a close resemblance to the random walk model.^78^ To conduct such statistical analysis, we follow Pant and Bharadwaj and Boyd; we construct “jump distance distributions” (JDD). The magnitude of the penetrant jump distance—*r*_p_(*i*)—is quantified by^14,79^
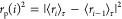
3In eq 3, *r_i_* is the position of the penetrant and τ is the time interval between jumps. JDD distributions are expected to show a positively skewed shape, suggesting short jumps and times of complete immobilization are the most frequent occurrences due to density fluctuations, obstructed motions, or complete constraint of the penetrant in the polymer matrix.^14,80^

**Figure 5:**
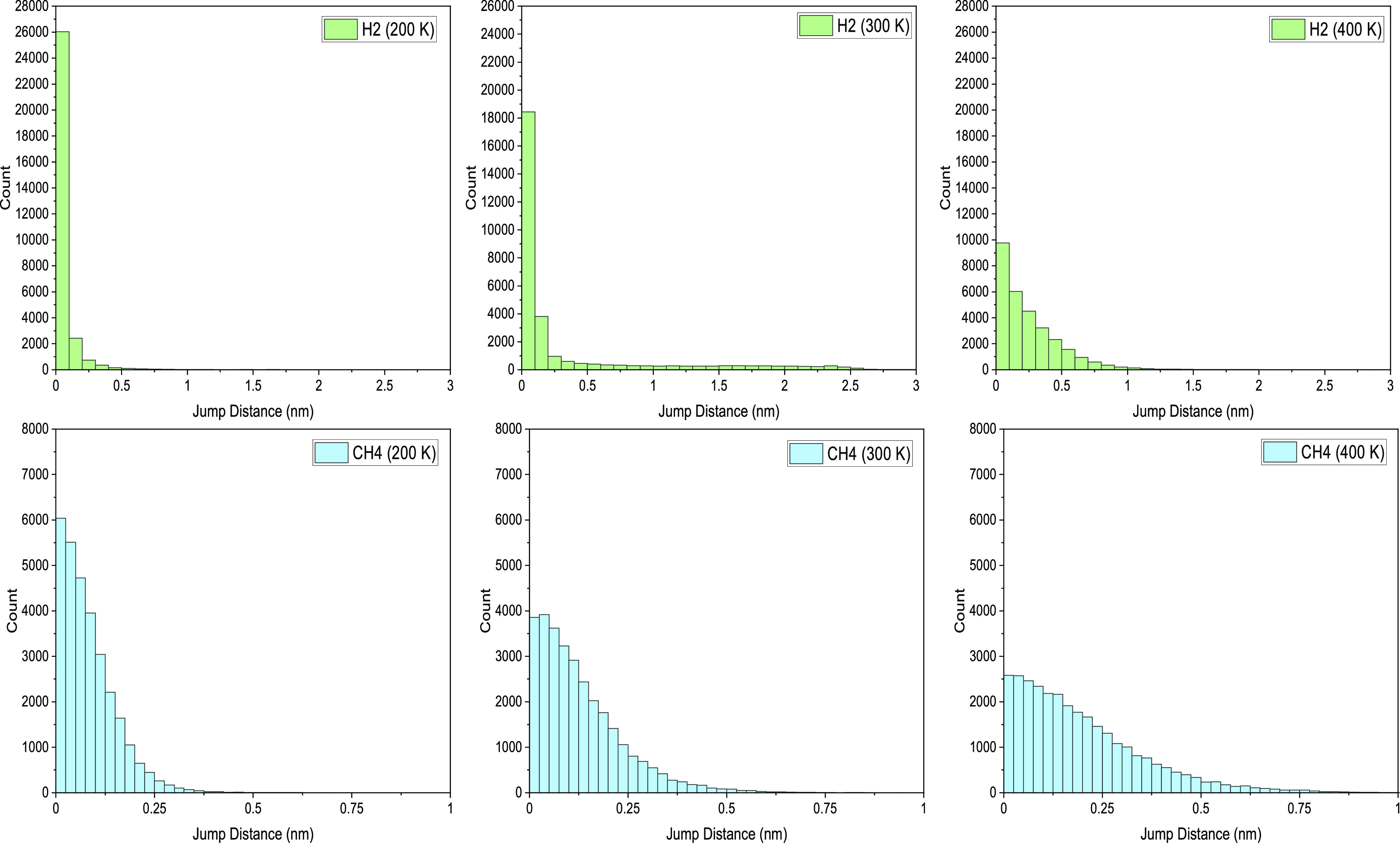
Jump distance distribution (JDD) observed for H_2_ and CH_4_ at 200, 300, and 400 K, over 30 ns simulation with a jump interval of 1 ps. Note that the scales are different in the various plots to reflect the different mobilities of the absorbed guest molecules.

In Figure 5, we report the JDDs for each system simulated. The results were calculated every 1 ps over the course of a 30 ns simulation. A time frame between movements of 1–5 ps is typically found to be a sufficient scale to observe jumps for small molecules.^16,49^

The immobility of methane at 200 K as seen in the trajectory map in Figure 2 yields the narrow JDD shown in Figure 5. In this plot, the vast majority of the CH_4_ jumps at 200 K are just above 0.05 nm, confirming that this gas molecule is likely bouncing in the low-density cavity it is occupying.

At 200 K, H_2_ favors shorter hops, though it can jump distances further than 0.69 nm, 3 times its molecular diameter, with more than half of its jumps successful transposed moves. CH_4_, however, spends much of its time bouncing within a single cavity. As the temperature increases, the frequency of shorter jumps (<0.1 nm), reduces substantially; however, the gas molecule is capable to make multiple large jumps (2.5 nm) at 300 K.

At 400 K, the JDD distribution has a larger, more even positively skewed spread, though not many particularly large jumps (around ∼1 nm). This is possibly due to the movement of the polymer chains. The matrix is largely motionless at low temperatures but its movements increase slightly at 300 K. This reduced but present movement of the polymer allows for more opportunity for fast-traveling H_2_ to make longer leaps in microtunnels being formed as they linger. These long jumps are somewhat obstructed as the temperature rises since the polymer chains have more energy to move more freely and microchannels change much more rapidly.

The jump length observed for CH_4_ increases with the increase in temperature, from under 0.25 nm at 200 K to 0.87 nm at 400 K. Further, displacement events of size close to 0 subside. As the atomic diameter of CH_4_ is 0.37 nm, clearly methane is mostly confined and does not transpose further than its molecular size at temperatures below 300 K, consistent with the trajectory map.

### 3.4 Relation between Polymer Structure and Guest Gas Diffusion

It is generally accepted that penetrant diffusion in semicrystalline polymers is regulated by the availability of free volume in amorphous regions, while the crystalline inclusions obstruct transport.^49,81^ Given the small size of molecular H_2_, it is of interest to inquire whether this expectation holds, as neither experiments nor simulations have yet probed this area in depth. The visualization of the simulation results discussed above highlights the possibility of long hops, which we found to occur in correspondence with the areas of aligned PE chains. Of note, Zhao recently noted that the orientation of branched polyethylene–graphene systems may contribute to the overall directional trajectory of small diatomic molecules such as hydrogen.^28^

**Figure 6:**
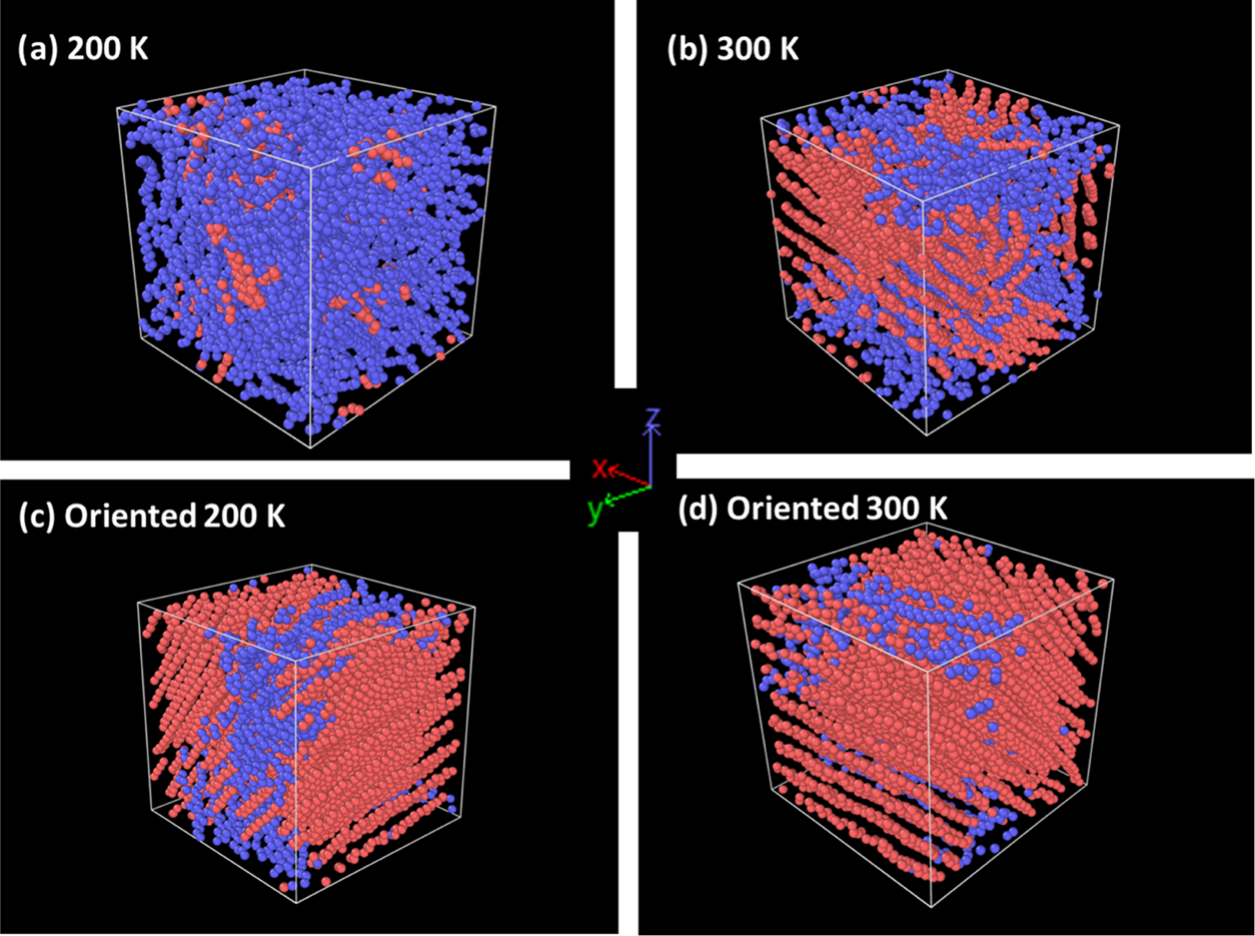
Structure of polyethylene systems: disordered (a, c) and ordered (b, d) at 200 and 300 K. The snapshots are visualized using the software Ovito. Each sphere represents one CH_2_ or CH_3_ united atom group in PE. The color code illustrates the relative order within the polymer matrix; red indicates that the chains show alignment and relative order, while blue indicates that the PE chains in these regions are disordered.

To answer this fundamental question, model PE ordered domains were created in our system by rapid cooling a PE melt.^82^ The initial 100 chain C_50_H_102_ system was cooled from 600 to 200 K over 500 ps, before heating it to 350 K for 30 ns, and then cooling it slowly to the desired temperatures over a further 10 ns of simulations. At the end of these cycles of annealing simulations, the PE chains were found to be aligned and partially oriented along the *X*–*Z* direction of the simulation box at both 200 and 300 K to varying degrees, as can be seen in Figure 6. It should be pointed out that these oriented systems were not used in any of the systems prior to this section.

The system contains regions where ordered “crystalline” (red region) and more disordered “amorphous” (blue region) formations coexist. The different PE regions display varying degrees of order, as can be distinctly appreciated in Figure 6. The order parameters^83^ or the degree of orientation in a system has been used as a measure of crystallinity in simulated systems and was used in this case to discriminate ordered and disordered regions.^84^ At 300 K (Figure 6d), the system was found to be approximately 68.5% crystalline or ordered which coincides with the degree of crystallinity expected for semicrystalline PE.^61^ In addition to this, the overall structure, as quantified using radial distribution functions, was found to be similar to what is expected of semicrystalline PE also. Further details of this analysis are reported in the Supporting Information (Part A).

As the primary objective of the ordered PE structure was to observe the effect of the chain alignment in the system; features such as loops, tails, and ties were not included as a focal point in the models. It is expected that these structures would affect the diffusion results because the latter are found to strongly depend on the relative orientation of next-neighbor PE chains.

**Figure 7:**
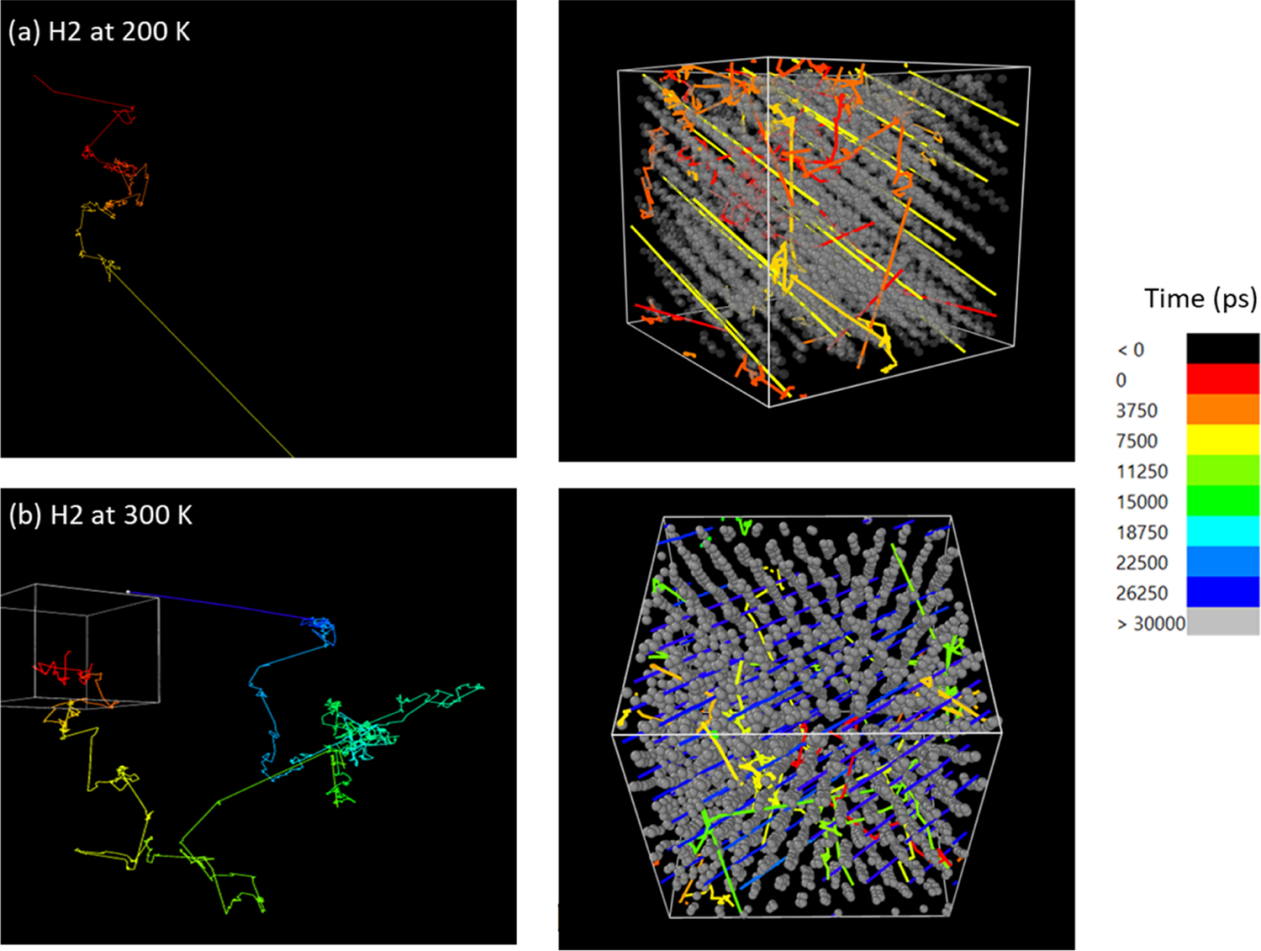
Trajectory of one H_2_ molecule absorbed within the oriented polyethylene (gray chains) system at 200 and 300 K. Visuals are prepared using the software Ovito.

Despite the guest molecules (either H_2_ or CH_4_) originally being placed in the disordered or amorphous region of the PE structures generated, the ordered domains were found to greatly influence the trajectory of both absorbed H_2_ and CH_4_ guest gases. The Open Visualization Tool, known as Ovito,^85^ was used to analyze the simulated trajectories. H_2_ at 200 K exhibited short clustered “cavity motions” followed by short jumps, like those discussed in amorphous PE (Figure 2). After 7.5 ns, the H_2_ molecule takes a much longer, nearly straight jump (see Figure 7). When analyzed in detail, this path was found to be composed of many small skipping jumps. It was also found that, instead of jumping through randomly occurring cavities in amorphous PE, H_2_ travels through the ordered PE, between and parallel to the aligned PE chains. Furthermore, our results suggest that H_2_ motion through these regions is undisturbed along very lengthy skipping motions until a more disordered region is explored by the diffusing gas molecule. As the dynamics of the polymer matrix is very slow at this temperature, below the *T*_g_, the ordered chains enable long jumps in the direction parallel to the polymer chains. This observation contradicts the notion that ordered domains entirely hinder gas transport of very small molecules and may have some influence upon the direction of flow.

**Figure 8:**
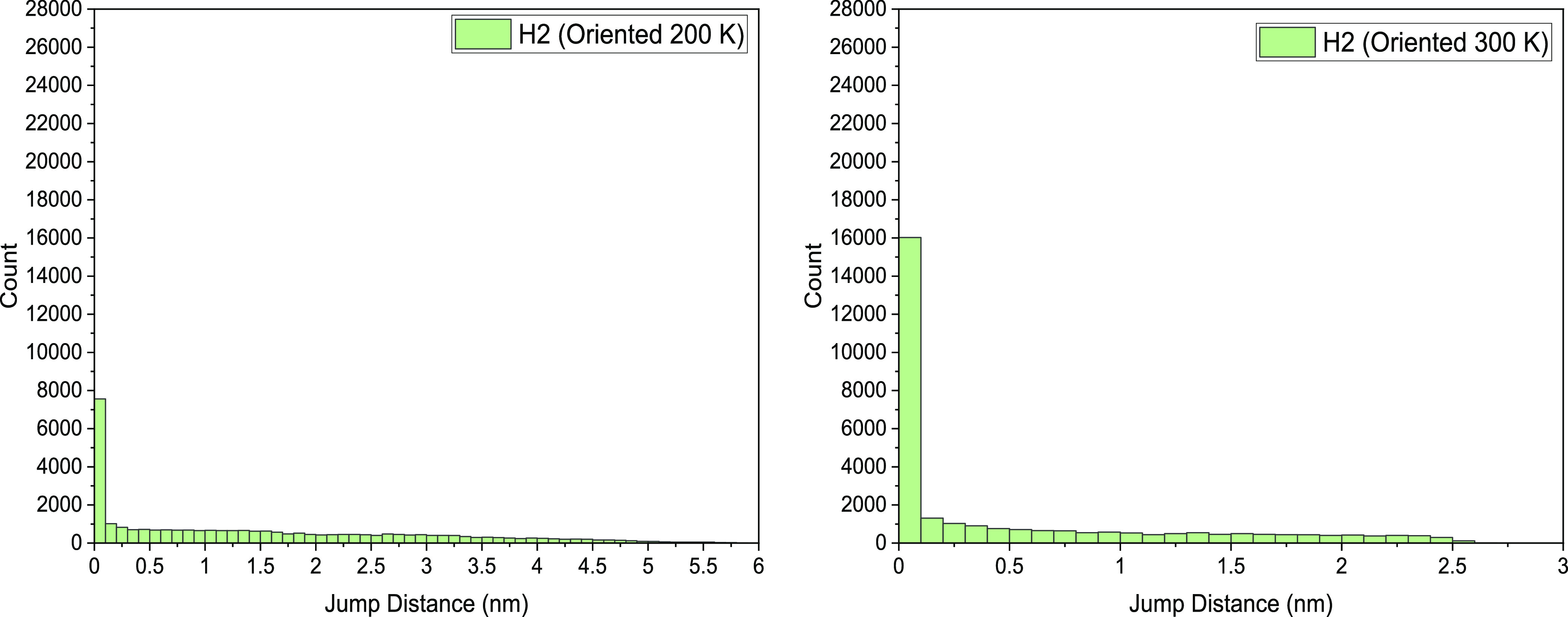
Jump distance distribution for H_2_ in ordered PE system at 200 and 300 K. Note the different extents of the *x* axis.

At 300 K, the polymer matrix maintains some alignment, but it also develops regions of disordered structures because *T* > *T*_g_. Nevertheless, though H_2_ hops between amorphous regions where free volume is available, as described above, after ∼26 ns, H_2_ shows long undisturbed “skips” along the PE chains, reflecting the mechanism described at 200 K. Though both penetrant molecules were placed in the disordered regions of the PE matrix at the beginning of these simulations, they were found to enter the ordered region at a point during the simulations, where their transport mechanism changed drastically. The longer skips can be clearly visualized in the jump distribution of H_2_ presented in Figure 8. In the oriented matrix, H_2_ displays a noticeably large range of jumps, both long and short, cresting at 5.8 nm. The jumps between 0.1 and 4.5 nm appear to be uniform, unlike the more positively skewed distribution seen in Figure 5. As can be viewed in the JDD of Figure 7, these long, undisturbed jumps correspond to the periods of time where the penetrant finds the straight path along the oriented chains. Instead of following a typical intra- and intercavity hopping motion or a fluid-like drifting motion with moving polymer chains, there is still a skipping motion along the alignment in the matrix. The ability to skip appears to be size-dependent also, as absorbed H_2_ was mostly mobile, whereas CH_4_ remains static within the ordered PE region.^14^

**Figure 9:**
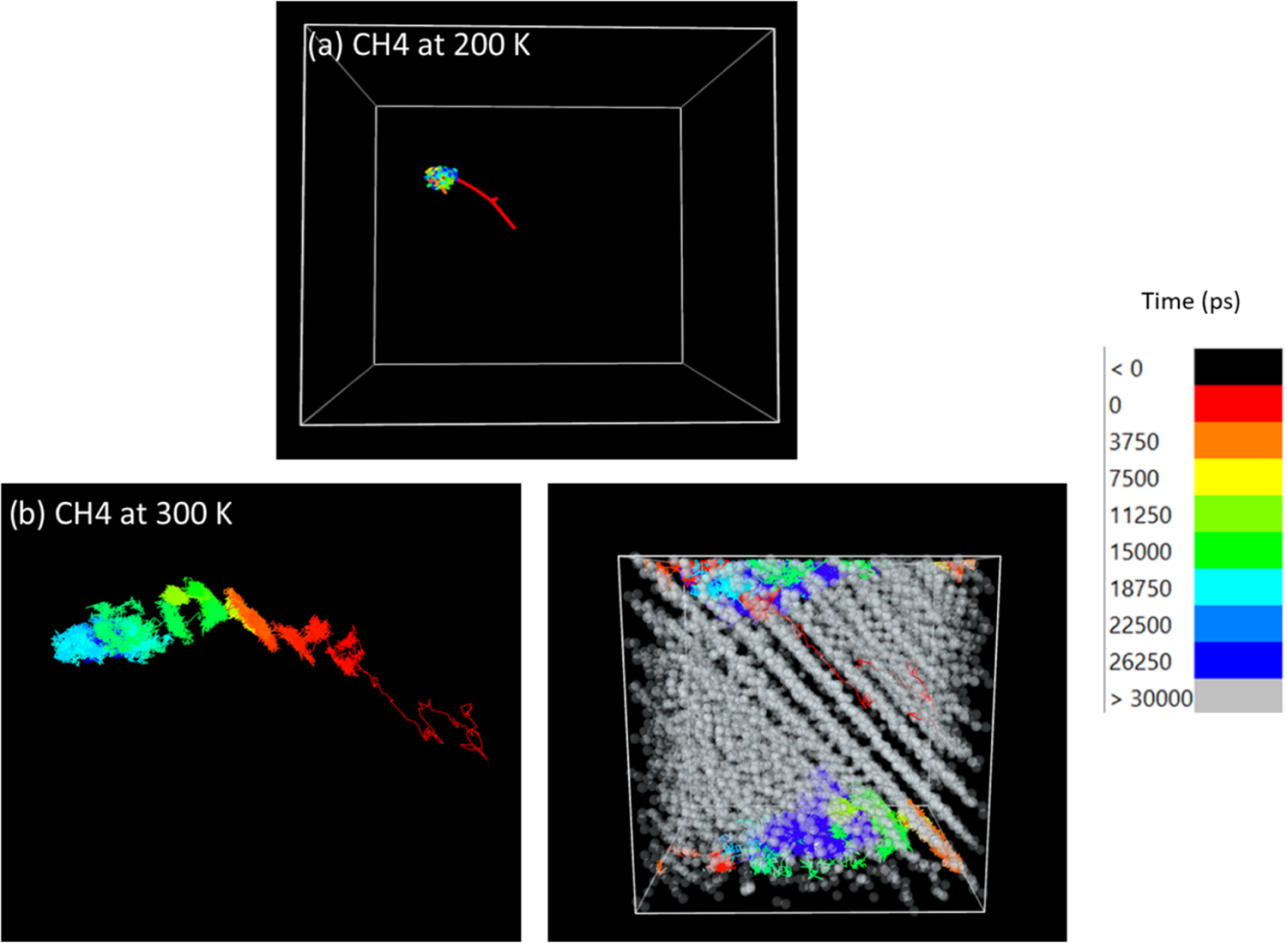
Trajectory of CH_4_ in an oriented polyethylene (gray chains) system at 200 and 300 K as observed using the Ovito visualization software.

The results shown in Figure 9a demonstrate that CH_4_ displays a path with features similar to those discussed for CH_4_ transport in disordered PE at 200 K (see Figure 2d). The penetrant remains clustered, contained in a singular cavity; however, when in the oriented PE, the molecule does appear to make a single brief hop along the alignment from its original position (red region) to the cavity it continues to occupy. This is expected as due to its size, CH_4_ cannot make longer skipping jumps in the same fashion as H_2_. At 300 K, CH_4_ has more freedom to move in the matrix; however, it only appears to make one slightly longer jump across the alignment to settle in a more disordered region of the PE matrix, which bears a greater semblance to amorphous PE. The guest gas molecule appears to be mostly contained by the central ordered PE region, with movement aligned with the PE chains. The direction of the polymer chains appears to have a lesser effect on methane compared to H_2_. Although its motion is constrained by the proximity between ordered PE chains, CH_4_ diffusion in this system appears to follow preferentially the alignment of the polymer chains.

These observations agree with the free volume theory of diffusion: as though H_2_ is able to briefly occupy the area between the oriented chains and travel in the *X*–*Z* direction, CH_4_ favors diffusing in the cavities of the more disordered and amorphous behaving regions.

**Figure 10:**
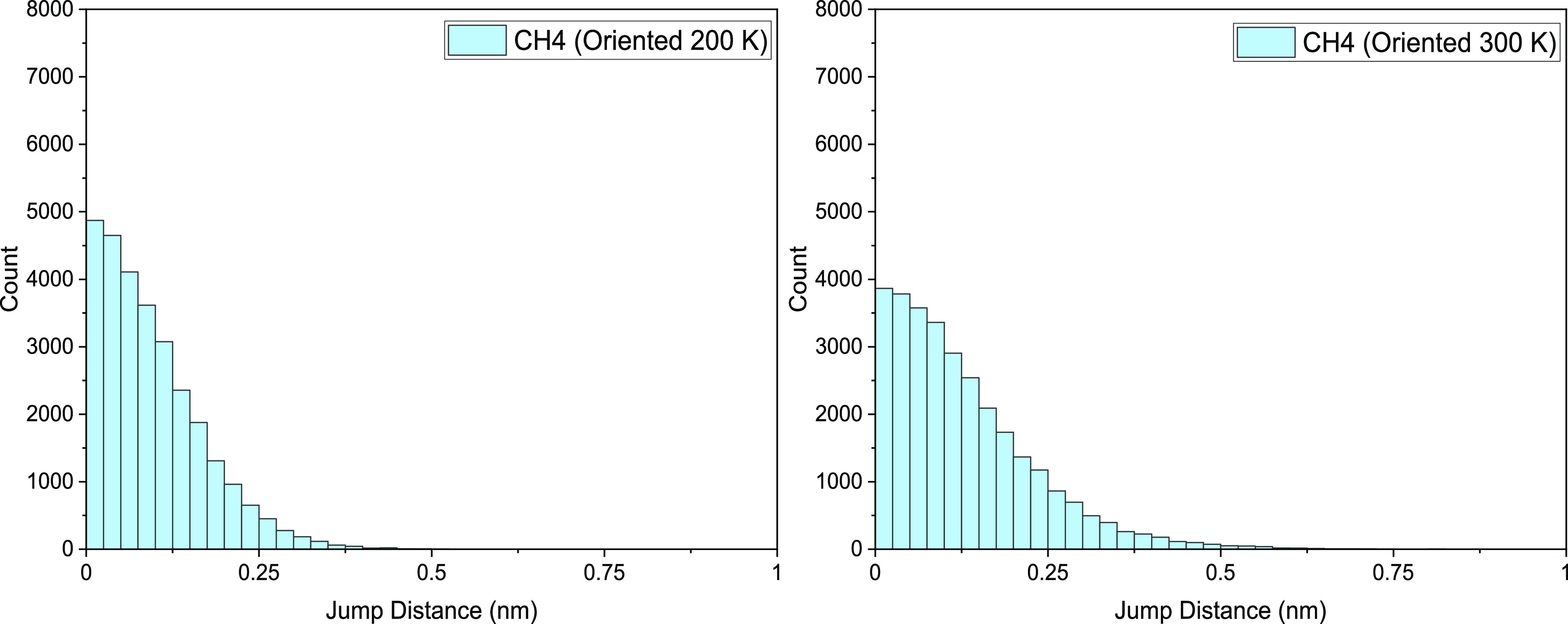
Jump distance distribution observed for CH_4_ in an ordered PE system at 200 and 300 K.

At 200 K, our results show that CH_4_ remains mostly immobile in both ordered and disordered PE matrices, although the jump distance reaches slightly longer values (0.46 nm) in disordered PE compared to results obtained in the ordered PE matrix. This is consistent with the expectation that crystallinity in material systems reduces the penetrant transport ability, as can be appreciated from the results shown in Figure 10.

As the temperature is increased to 300 K, the overall JDD is rather similar, although the amount of very short jumps (<0.1 nm) slightly reduced in the ordered system at both temperatures. This is possibly due to CH_4_ being contained by narrow cavities formed by the aligned polymer chains, allowing for slightly more movement than the full immobilization in the disordered system cavities seen prior. The jump distance very marginally increases in the disordered system to a maximum value of 0.74 nm from 0.69 nm in the ordered system. This suggests that although the polymer matrix restrains the movement of CH_4_ under both conditions, this is slightly more apparent in the oriented system.

Despite methane appearing to be very much similarly contained in a system with both “semicrystalline” or amorphous characteristics, the orientation in this system appears to contain the molecule more effectively to specific areas. H_2_, however, displays the potential to utilize oriented structures within the PE polymer matrix to orchestrate relatively long, undisturbed jumps at temperatures below the polymer *T*_g_. This is accomplished by a long skipping jump motion, with the guest particle moving along “ordered pathways”; much like a “tunnel” or “highway”. These results suggest that the effectiveness of gas containment may be controlled considerably by the polymer manufacturing process, as this can affect the order within the polymer matrix.

## 4 Conclusions

In pursuit of building a better understanding of hydrogen gas transport and containment in polyethylene, the diffusive behaviors and jump mechanisms of both hydrogen and methane were studied in a bulk polyethylene polymer matrix.

The diffusion coefficient obtained from the atomistic simulations conducted here was found to be consistent with experimental and computational reports in the literature, with H_2_ diffusing considerably faster than CH_4_ due to the differences in their molecular sizes and varying interactions with the polymer matrix. While for CH_4_ our results show that the PE glass transition temperature strongly affects the diffusion of the guest gas molecule due to the increased mobility of the PE chains at temperatures above *T*_g_, our results show that the mobility of the PE chains has little effect on the diffusion coefficient for the absorbed H_2_ molecule, which effectively diffuses through the polymer matrix. This is a consequence of the much smaller molecular size of H_2_ than CH_4_ compared to the size of the molecular cavities formed within the amorphous PE matrix. To confirm that size differences, as opposed to differences in polymer–guest interactions were responsible for the observed differences between CH_4_ and H_2_ diffusion in the PE matrix, one additional simulation was conducted at 300 K. In this simulation, one methane-like molecule was modeled via the Weeks, Chandler, and Andersen (WCA) force field.^86^ In this method, the attractive portion of the interaction potentials was removed, allowing us to quantify the effect of molecular size on the results. The simulation results obtained for the WCA methane were consistent with those reported for CH_4_ in the remainder of the manuscript, as described in detail in the Supporting Information, Part B. This strengthens the hypothesis that molecular size is the main differentiator for the behavior of CH_4_ vs H_2_ confined in amorphous PE matrixes.

The transport mechanisms identified for the absorbed gas molecules via analysis of simulation trajectories were: intracavity hopping jump motions between low-density polymer “cages”, drifting jump motions in a more mobile amorphous system, and relatively long skipping jump motions through chain alignments. Where methane mainly presented hopping and drifting motion and was very restricted by the polymer matrix, hydrogen displayed all three motion mechanisms. Methane typically favored drifting motions in the amorphous PE system at 400 K and hopping motions at 300 K, whereas hydrogen favors long fast “hops” at all temperatures in addition to engaging in clustered skips at points of order in the chains. This suggests that despite the ease with which H_2_ can diffuse through polymer matrices, the orientation of the polymer matrix may strongly influence its containment capabilities. This implies that the manufacturing process, such as injection, extrusion, or compression molding, may have strong effects on the ability of PE to function as a containment material, and more so for H_2_ than for larger guest molecules such as CH_4_.

## Supporting Information Available

Additional details of simulation results, methodology for assessing the degree of crystallinity or order in polymer matrix system, radial distribution function data, and additional simulation results for system modeled with the WCA force field further investigating the effect of the penetrant size (PDF)
