# Supplementary material for: Hop, Skip, and Jump: Hydrogen Molecular Transport through Amorphous Polyethylene Matrices Studied via Molecular Dynamics Simulations
Source: Ind Eng Chem Res. 2023 Nov 1;62(46):19893–906. doi: 10.1021/acs.iecr.3c02213 (PMC10682999; doi:10.1021/acs.iecr.3c02213)
Supplement: Supplementary file 1 — ie3c02213_si_001.pdf [file ie3c02213_si_001.pdf]

Supporting Information

A Hop, a Skip, and a Jump: Hydrogen Molecular Transport through Amorphous Polyethylene Matrices Studied via Molecular Dynamics Simulations

Candice Divine-Ayela[1], Felipe Perez [2], Alberto Striolo[1,2 *]

[1]Department of Chemical Engineering, University College London, London, WC1E 6BT, United Kingdom

[2]School of Sustainable Chemical, Biological and Materials Engineering, The University of Oklahoma, Oklahoma United States

*Corresponding author e-mail: astriolo@ou.edu

Part A. Ordered, Semicrystalline Systems

RDF Analysis

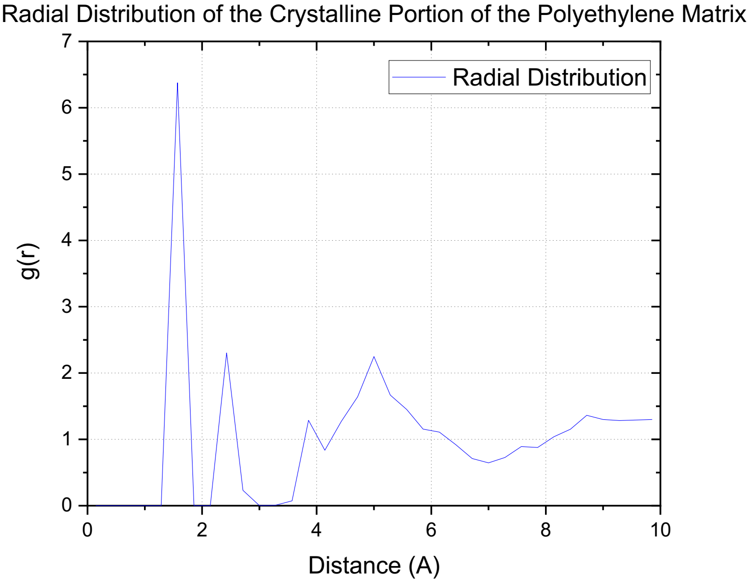

Figure 1 Radial Distribution of the crystalline region of the Polyethylene region at 300 K

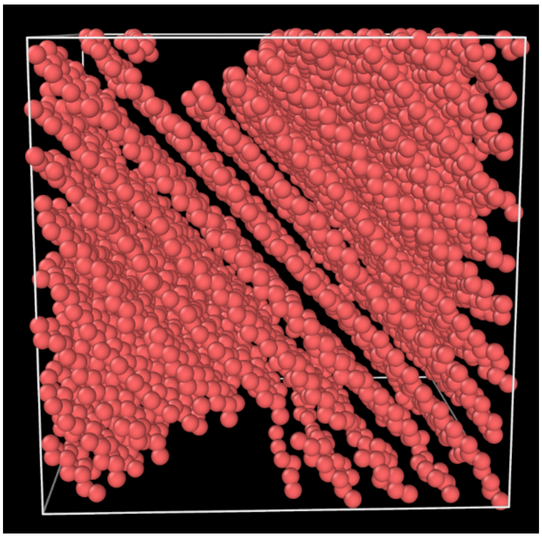

Figure 2 The crystalline region of the PE system (red) with the disordered (Amorphous) regions removed. Visualized and calculated using Ovito Software.

The radial distribution of the ordered polyethylene system at new room temperature aligns with the core silhouette of what is expected of pristine polyethylene. [1] The first peak, located at approximately 1.54

angstroms, coincides with the carbon-carbon interactions. This peak is higher and narrower than the second peak, indicating that the carbons are closely packed together.

Order parameter

We calculated the global order parameter P_2 for every configuration at different values of temperature. The global order parameter is given by:

$$P_2 = \left\langle \frac{3cos^2\psi - 1}{2} \right\rangle$$

where ψ is the angle between two chord vectors and angular brackets $\langle \quad \rangle$ denote average over all pairs of chord vectors. A chord vector is the vector that connects the centers of two adjacent bonds along the polymer chain (Fujiwara and Sato, 2001). [2] The global order parameter takes values between 1.0 (all chord vectors are perfectly aligned) and 0.0 (chord vectors are oriented randomly in the system).

We also calculated the local order parameter $P_{2,i}$ of each bead i in the system. In this case, the average presented in the equation above is calculated using only chord vectors that correspond to beads within a certain cutoff away from bead i. In our case, the cutoff is $2.5\sigma = 10$ Å. To determine whether a bead is considered as crystalline or amorphous, a threshold value of 0.40 was used Yan et al. (2021). [3] Thus, if $P_{2,i} > 0.40$, bead i is considered to be part of a crystalline neighborhood, otherwise it is part of an amorphous environment. The end beads of every polymer chain were assigned the same crystallinity value given to their bonding bead. This can be visualized in Figure 3.

Table 1. Calculated Degree of crystallinity for each Polyethylene at each temperature condition.

System Temperature (K)	Degree of Crystallinity %
200 (Disordered)	0.85
200 (Ordered)	60.47
300 (Disordered)	21.44
300 (Ordered)	68.49
400	0.024

The local bond order parameter of the chains within the system was utilized to determine the order of the system. This approach, which has been utilized in previous studies to identify crystallinity in MD. [2] When conducting experiments, it is expected that HDPE will have a degree of crystallinity ranging from 51% to 90%, with an average density of 954 kg/m^3 [4] at standard conditions. This falls

within the spectrum of fully amorphous (below 850 kg/m^3) and fully crystalline (1000 kg/m^3). Our own computational results align with this finding. [5] [6] [7]

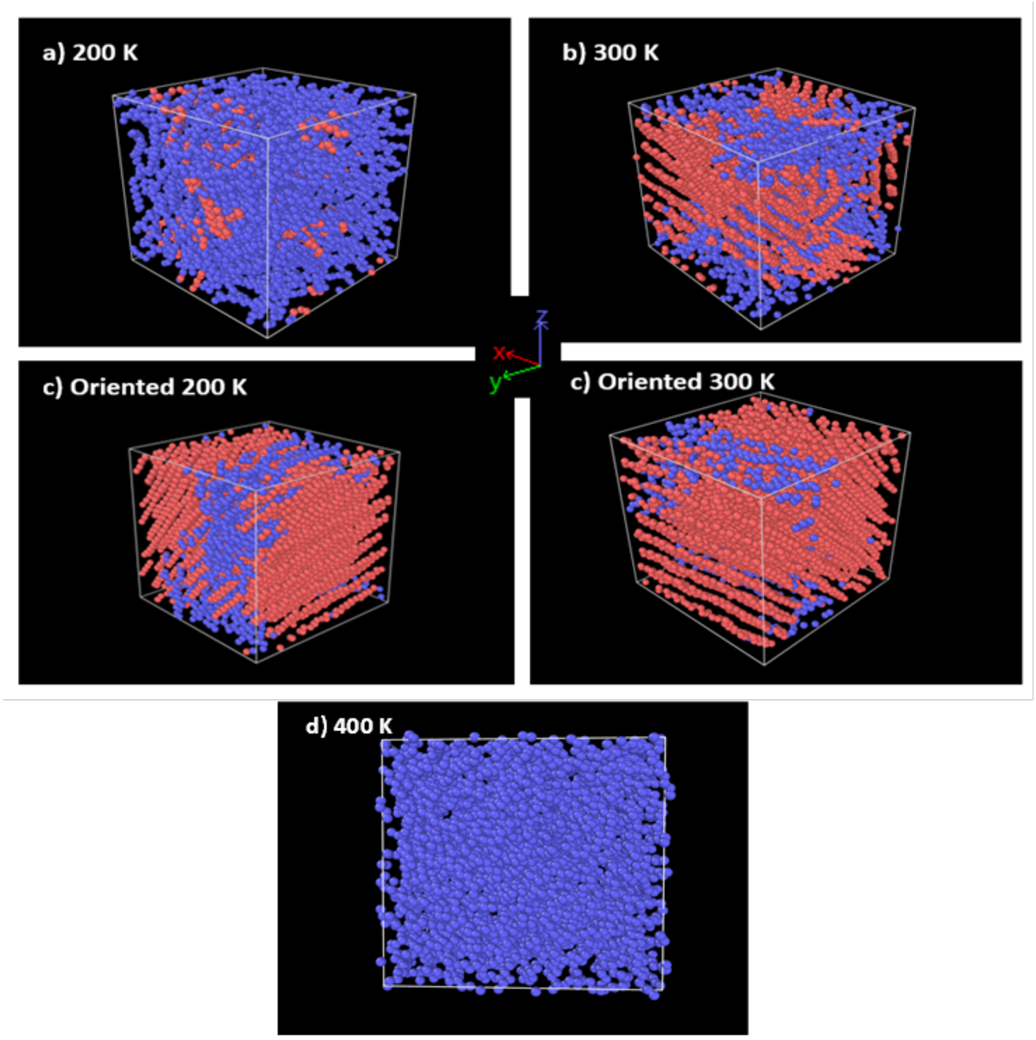

Figure 3 Polyethylene matrices with the crystalline or ordered regions indicated in red and the disordered, amorphous regions indicated in blue visualised using Ovito.

Part B: WCA Particle

In a study conducted by Weeks et al. [8] (WCA) over twenty-five years ago, a fluid consisting of interacting particles governed by the 6-12 Lennard-Jones (LJ) potential was examined. The potential was truncated at its minimum and shifted to zero at the cut-off distance, resembling a "hard sphere" reference system. This type of interaction, featuring solely repulsive forces, is commonly known as the WCA potential. In this way, the path of the methane could be further examined and confirm that the chosen path was more influence by the

penetrant size. As can be viewed in Figures 4A and 4B, the particle exhibits similar behaviours to that of CH_4 with full LJ potential and appears to be limited predominantly by size as theorised.

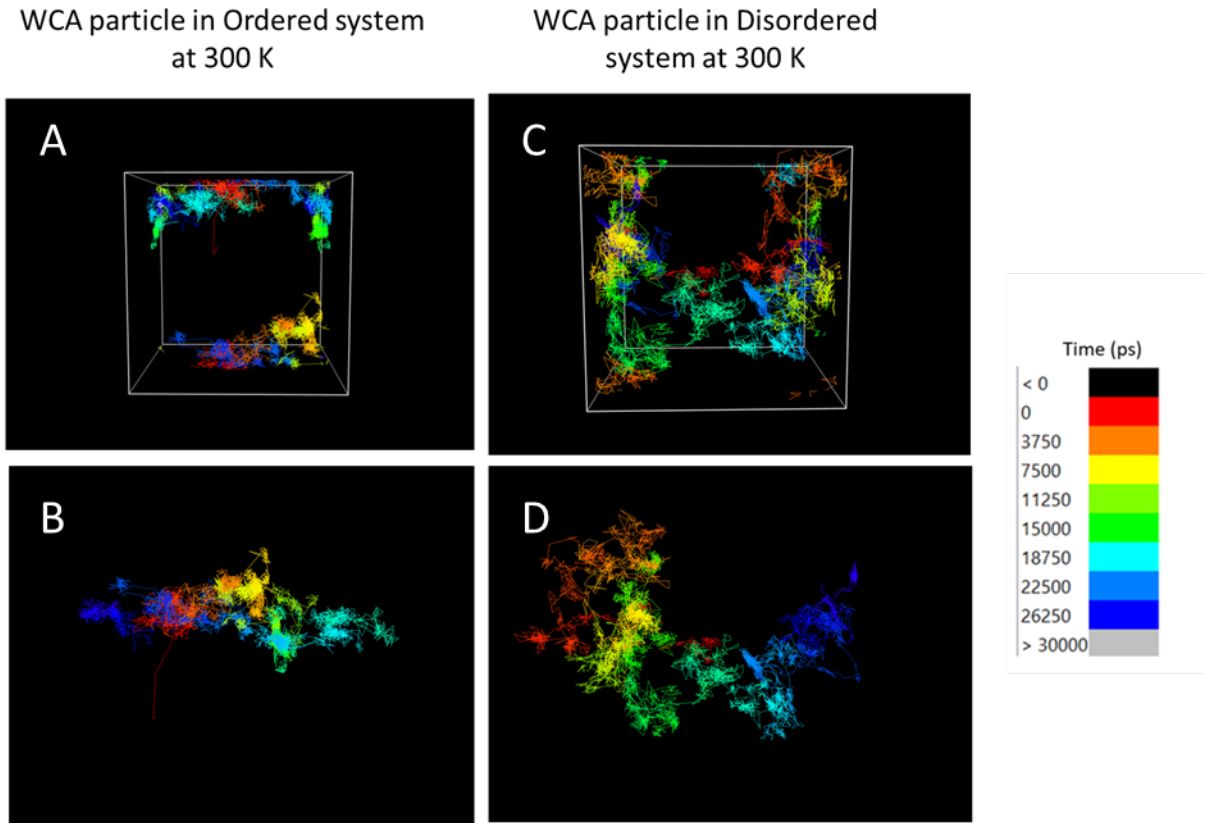

Figure 4. Both the boxed and unwrapped trajectory of a WCA particle at 300 K in the ordered system (A and B) and in the disordered system (C and D).

References

1. Su, C.-H., et al., *The Mechanical Behaviors of Polyethylene/Silver Nanoparticle Composites: an Insight from Molecular Dynamics study.* Scientific Reports, 2020. **10**(1): p. 7600.
2. Fujiwara, S. and T. Sato, *Molecular dynamics study of structure formation of a single polymer chain by cooling.* Computer Physics Communications, 2001. **142**(1): p. 123-126.
3. Yan, Z., A. Zaoui, and F. Zaïri, *Crystallization and mechanical behavior of semi-crystalline polyethylene.* Physica Scripta, 2021. **96**(12): p. 125729.
4. Li, D., et al. *Effect of Crystallinity of Polyethylene with Different Densities on Breakdown Strength and Conductance Property*. Materials, 2019. **12**, DOI: 10.3390/ma12111746.
5. Brayton, A.L., et al., *Vibrational Analysis of Semicrystalline Polyethylene Using Molecular Dynamics Simulation.* Macromolecules, 2017. **50**(17): p. 6690-6701.
6. Piggot, T.J., et al., *On the Calculation of Acyl Chain Order Parameters from Lipid Simulations.* Journal of Chemical Theory and Computation, 2017. **13**(11): p. 5683-5696.
7. Yuan, Z. and X.-R. Xu, *Chapter Six - Surface characteristics and biotoxicity of airborne microplastics*, in *Comprehensive Analytical Chemistry*, J. Wang, Editor. 2023, Elsevier. p. 117-164.
8. Weeks, J.D., D. Chandler, and H.C. Andersen, *Role of repulsive forces in determining the equilibrium structure of simple liquids.* The Journal of Chemical Physics, 1971. **54**(12): p. 5237-5247.
